# Do we have enough evidence to use chloroquine/hydroxychloroquine as a public health panacea for COVID-19?

**DOI:** 10.6061/clinics/2020/e1928

**Published:** 2020-04-30

**Authors:** Vitória Andrade Palmeira, Larissa Braga Costa, Lucas Giandoni Perez, Victor Teatini Ribeiro, Katharina Lanza, Ana Cristina Simões e Silva

**Affiliations:** Laboratorio Interdisciplinar de Investigacao Medica, Faculdade de Medicina, Universidade Federal de Minas Gerais (UFMG), Belo Horizonte, MG, BR

The severe acute respiratory syndrome coronavirus 2 (SARS-CoV-2), a novel virus, was emerged in Wuhan, China, in December 2019. On March 11^th^, the World Health Organization (WHO) declared the outbreak of coronavirus disease 2019, and termed “COVID-19,” a pandemic. At that point, there were 118,000 confirmed cases and 4,291 victims worldwide 1. Since then, researchers around the world have been analyzing the genetic structure, multiplication patterns and the cellular mechanisms of SARS-CoV-2 *in vitro* and *in vivo* to identify possible drug targets 2.

Four months after the onset of the outbreak, physicians from different countries and laboratories have proposed several empirical treatments for COVID-19. To date, there are over 335 COVID-19–related clinical trials registered on the National Institutes of Health (NIH) website 3. The proposed drugs aim to prevent viral entry into host cells, interrupt viral replication, and attenuate virus-induced inflammatory responses. Examples of the drugs being tested for each of these mechanisms are recombinant human angiotensin converting enzyme 2 (RhACE2), the antiviral remdesivir, and the immunosuppressant, tocilizumab, respectively.

However, the most promising drugs appear to be old drugs, the antimalarial and immunomodulatory medication, chloroquine and its hydroxy analog, hydroxychloroquine. Chloroquine and hydroxychloroquine are by far the most popular drugs proposed for treatment and prophylaxis, appearing in 97 of the COVID-19 clinical trials registered on the NIH site. These aminoquinolines, which were discovered in 1934 and are inexpensively produced in several countries, and have well-known pharmacokinetic and pharmacodynamic properties. The antiviral mechanisms of chloroquine are based on its capacity to increase the endosomal pH. This prevents enveloped viruses, such as those belonging to the *Coronaviridae* family (*e.g.*, SARS-CoV-2), from entering and releasing their genetic material into the host cells and from replicating their envelopes. Furthermore, in severe and complicated COVID-19 cases, the anti-inflammatory effects of chloroquine/hydroxychloroquine may also be of importance, as both medications can suppress the production and release of tumor necrosis factor (TNF) and interleukin 6 (IL-6) 4.

While the mechanisms of action of chloroquine/hydroxychloroquine are well-established, so are the side effects. Serious retinopathies and cardiopathies associated with bioaccumulation of the drugs are described in literature 5. One study at the Clinics Hospital of the University of São Paulo evaluated 350 patients with systemic lupus erythematosus who were treated with chloroquine. The prevalence of side effects was 35.7%, with the most common being ocular alterations (17%), followed by gastrointestinal symptoms (10%), and dermatological (3.4%), neuromuscular (1.7%), and psychiatric alterations (0.3%) 6. In Brazil, a phase 2 clinical trial on COVID-19 sponsored by the state of Amazonas was suspended after 25% of patients developed QT prolongation (>500 m/s) owing to cardiotoxicity 7.

Although chloroquine/hydroxychloroquine might yield promising results, they should not be announced as a cure by politicians and the mass media, as they have never been tested as treatments for *Coronaviridae* viruses. Clinical trials are ongoing during the course of this pandemic, yet no scientific evidence has been found to support the widespread use of these medications. Table 1 lists a few trials in advanced phases that have randomized allocation, double masking, and significant numbers of proposed participants. However, none of them have reported results as of yet.

Recent declarations by the President of Brazil recommending the indiscriminate use of chloroquine resulted in a shortage of the drug in several cities 8. Aside from the lack of evidence supporting his proclamations, the president's action hampered the treatment of patients who truly need chloroquine for systemic lupus erythematosus and rheumatoid arthritis. Pronouncements by the French president also appeared to have pressurized French doctors to prescribe the drug, despite its unconfirmed efficacy and many possible side effects 9. The same situation occurred in the United States (USA); the country's president has endorsed chloroquine in a highly politicized debate over its use 10.

A serious health system should not encourage protocols based on political beliefs or case reports. Scientific trial phases exist and are in constant review so we can guarantee some basic level of safety regarding prescription medications. Ignoring or skipping these phases can result in manmade medical disasters, such as the thalidomide-induced teratogenesis tragedies in the 20th century, which resulted in more than 10,000 children were born with debilitating malformations, leading to the scientific community being forced to rethink interventional studies and the approval process for new drugs 11. Drug repositioning is possible and encouraged in case of pandemics, but chloroquine/hydroxychloroquine should not be recommended to the general population as if these medications were supported by grade 1a evidence 12. Affirmations of the drugs' effectiveness, such as those by some of our world leaders in the middle of the pandemic will only spread fear and the erroneous belief in chloroquine/hydroxychloroquine as a panacea.

In summary, there are still no effective treatments for COVID-19. Though chloroquine/hydroxychloroquine may be promising, their use should be restricted to ongoing clinical trials until we have enough evidence to recommend it to the general population. We must consider the long-term consequences of chloroquine/hydroxychloroquine administration and respect the scientific approach. A well-established, evidence-based health care policy may save more lives than a swift implementation of unsupported recommendations.

## Figures and Tables

**Table 1:** Ongoing randomized, double-blind clinical trials on the therapeutic and prophylactic use of chloroquine/hydroxychloroquine in COVID-19 patients.

	NCT NUMBER	COUNTRY	INTERVENTION	STUDY PHASE	NUMBER ENROLLED
Clinical Trials for Treatment of COVID19	NCT04342221	Germany	Hydroxychloroquine	Phase 3	220
	NCT04329923	United States	Hydroxychloroquine	Phase 2	400
	NCT04340544	Germany	Hydroxychloroquine	Phase 3	2700
	NCT04333654	United States	Hydroxychloroquine and SAR321068	Phase 1	210
	NCT04315896	Mexico	Hydroxychloroquine	Phase 3	500
	NCT04308668	United States	Hydroxychloroquine	Phase 3	3000
	NCT04329611	Canada	Hydroxychloroquine	Phase 3	1660
	NCT04332991	United States	Hydroxychloroquine	Phase 3	510
	NCT04325893	France	Hydroxychloroquine	Phase 3	1300
	NCT04331834	Spain	Hydroxychloroquine	Phase 3	440
Clinical Trials for Prophylaxis of COVID19	NCT04334928	Spain	Emtricitabine/Tenofovir Disoproxil, Hydroxychloroquine and Placebo	Phase 3	4000
	NCT04341441	United States	Hydroxychloroquine	Phase 3	3000
	NCT04336748	Austria	Hydroxychloroquine	Phase 3	440
	NCT04334148	United States	Hydroxychloroquine and Placebo	Phase 3	15000
	NCT04328467	United States	Hydroxychloroquine	Phase 3	3500
	NCT04328285	France	Hydroxychloroquine, Lopinavir/Ritonavir and Placebo	Phase 3	1200
	NCT04303507	United States	Chloroquine/Hydroxychloroquine	Not applicable	40000

Legend: COVID-19 = coronavirus disease 2019; NTC= National Clinical Trial.
